# Supplementary material for: Male applicants are more likely to be awarded fellowships than female applicants: A case study of a Japanese national funding agency
Source: PLoS One. 2023 Oct 25;18(10):e0291372. doi: 10.1371/journal.pone.0291372 (PMC10599527; doi:10.1371/journal.pone.0291372)
Supplement: S1 File — (PDF) [file pone.0291372.s002.pdf]

Supplementary results:

Male applicants are more likely to be awarded fellowships than female applicants: a case study of a Japanese national funding agency

Daisuke Kyogoku and Yoko Wada

Table S1. The overall gender gap among Overseas applicants. *P* values are of Wald *z* tests. SE and SD stand for standard error and standard deviation, respectively.

Variable	Estimate (SE)	*z* value	*P* value
(Intercept)	−1.597 (0.0992)	−16.10	< 0.0012
Gender (male)	0.400 (0.106)	3.214	0.001
Random effect			
Year (SD)	0.0533		

Table S2. The overall gender gap among RPD applicants. *P* values are of Wald *z* tests. SE and SD stand for standard error and standard deviation, respectively.

Variable	Estimate (SE)	*z* value	*P* value
(Intercept)	−1.063 (0.0639)	−16.64	< 0.001
Gender (male)	0.270 (0.254)	1.064	0.287
Random effect			
Year (SD)	< 0.001		

Table S3. The overall gender gap among PD applicants. *P* values are of Wald *z* tests. SE and SD stand for standard error and standard deviation, respectively.

Variable	Estimate (SE)	*z* value	*P* value
(Intercept)	−1.776 (0.0978)	−18.15	< 0.001
Gender (male)	0.196 (0.0607)	3.231	0.001
Random effect			
Year (SD)	0.185		

Table S4. The overall gender gap among DC2 applicants. *P* values are of Wald *z* tests. SE and SD stand for standard error and standard deviation, respectively.

Variable	Estimate (SE)	*z* value	*P* value
(Intercept)	−1.509 (0.0305)	−49.48	< 0.001
Gender (male)	0.160 (0.0351)	4.549	< 0.001
Random effect			
Year (SD)	< 0.001		

Table S5. The overall gender gap among DC1 applicants. *P* values are of Wald *z* tests. SE and SD stand for standard error and standard deviation, respectively.

Variable	Estimate (SE)	*z* value	*P* value
(Intercept)	−1.478 (0.0392)	−37.71	< 0.001
Gender (male)	0.123 (0.0447)	2.754	0.006
Random effect			
Year (SD)	< 0.001		

Table S6. The correlation between the gender gap and the number of female applications among PD applicants. *P* values are of Wald *z* tests. SE and SD stand for standard error and standard deviation, respectively.

Variable	Estimate (SE)	*z* value	*P* value
(Intercept)	0.658 (0.152)	4.318	< 0.001
Number of female applicants	−0.005 (0.002)	−2.679	0.007
Random effect			
Year (SD)	< 0.001		
Research field (SD)	0.188		

Table S7. The correlation between the gender gap and the proportion of female applications among PD applicants. *P* values are of Wald *z* tests. SE and SD stand for standard error and standard deviation, respectively.

Variable	Estimate (SE)	*z* value	*P* value
(Intercept)	1.144 (0.173)	6.595939	< 0.001
Proportion of female applicants	−2.842 (0.533)	−5.3276	< 0.001
Random effect			
Research field (SD)	0.010		

Table S8. The correlation between the gender gap and the number of male applications among PD applicants. *P* values are of Wald *z* tests. SE and SD stand for standard error and standard deviation, respectively.

Variable	Estimate (SE)	*z* value	*P* value
(Intercept)	0.163 (0.269)	0.606	0.544
Number of male applicants	0.001 (0.001)	0.727	0.467
Random effect			
Year (SD)	0.003		
Research field (SD)	0.057		

Table S9. The correlation between the gender gap and the number of female applications among Overseas applicants. *P* values are of Wald *z* tests. SE and SD stand for standard error and standard deviation, respectively.

Variable	Estimate (SE)	*z* value	*P* value
(Intercept)	−0.778 (0.486)	−1.600	0.110
Number of female applicants	0.034 (0.021)	1.637	0.102
Random effect			
Year (SD)	0.012		
Research field (SD)	0.634		

Table S10. The correlation between the gender gap and the proportion of female applications among Overseas applicants. *P* values are of Wald *z* tests. SE and SD stand for standard error and standard deviation, respectively.

Variable	Estimate (SE)	*z* value	*P* value
(Intercept)	−0.843 (0.662)	−1.274	0.203
Proportion of female applicants	2.800 (2.401)	1.166	0.243
Random effect			
Year (SD)	< 0.001		
Research field (SD)	0.958		

Table S11. The correlation between the gender gap and the number of male applications among Overseas applicants. *P* values are of Wald *z* tests. SE and SD stand for standard error and standard deviation, respectively.

Variable	Estimate (SE)	*z* value	*P* value
(Intercept)	−0.639 (0.534)	−1.196	0.232
Number of male applicants	0.006 (0.005)	1.131	0.258
Random effect			
Year (SD)	< 0.001		
Research field (SD)	0.949		

Table S12. The correlation between the gender gap and the number of female applications among DC2 applicants. *P* values are of Wald *z* tests. SE and SD stand for standard error and standard deviation, respectively.

Variable	Estimate (SE)	*z* value	*P* value
(Intercept)	0.116 (0.133)	0.873	0.383
Number of female applicants	0.0003 (0.0007)	0.397	0.692
Random effect			
Year (SD)	0.061		
Research field (SD)	0.160		

Table S13. The correlation between the gender gap and the proportion of female applications among DC2 applicants. *P* values are of Wald *z* tests. SE and SD stand for standard error and standard deviation, respectively.

Variable	Estimate (SE)	*z* value	*P* value
(Intercept)	0.039 (0.143)	0.274	0.784
Proportion of female applicants	0.444 (0.456)	0.974	0.330
Random effect			
Year (SD)	0.060		
Research field (SD)	0.161		

Table S14. The correlation between the gender gap and the number of male applications among DC2 applicants. *P* values are of Wald *z* tests. SE and SD stand for standard error and standard deviation, respectively.

Variable	Estimate (SE)	*z* value	*P* value
(Intercept)	0.422 (0.127)	3.324	< 0.001
Number of male applicants	−0.0006 (0.0002)	−2.327	0.020
Random effect			
Year (SD)	0.056		
Research field (SD)	0.126		

Table S15. The correlation between the gender gap and the number of female applications among DC1 applicants. *P* values are of Wald *t* tests. SE and SD stand for standard error and standard deviation, respectively.

Variable	Estimate (SE)	*t* value	*P* value
(Intercept)	−0.110 (0.122)	−0.902	0.376
Number of female applicants	0.003 (0.001)	2.408	0.024

Table S16. The correlation between the gender gap and the proportion of female applications among DC1 applicants. *P* values are of Wald *z* tests. SE and SD stand for standard error and standard deviation, respectively.

Variable	Estimate (SE)	*z* value	*P* value
(Intercept)	−0.096 (0.059)	−1.636	0.102
Proportion of female applicants	0.959 (0.199)	4.821	< 0.001
Random effect			
Year (SD)	< 0.001		
Research field (SD)	< 0.001		

Table S17. The correlation between the gender gap and the number of male applications among DC1 applicants. *P* values are of Wald *z* tests. SE and SD stand for standard error and standard deviation, respectively.

Variable	Estimate (SE)	*z* value	*P* value
(Intercept)	0.349 (0.093)	3.763	< 0.001
Number of male applicants	−0.0006 (0.0003)	−2.185	0.029
Random effect			
Research field (SD)	0.069		

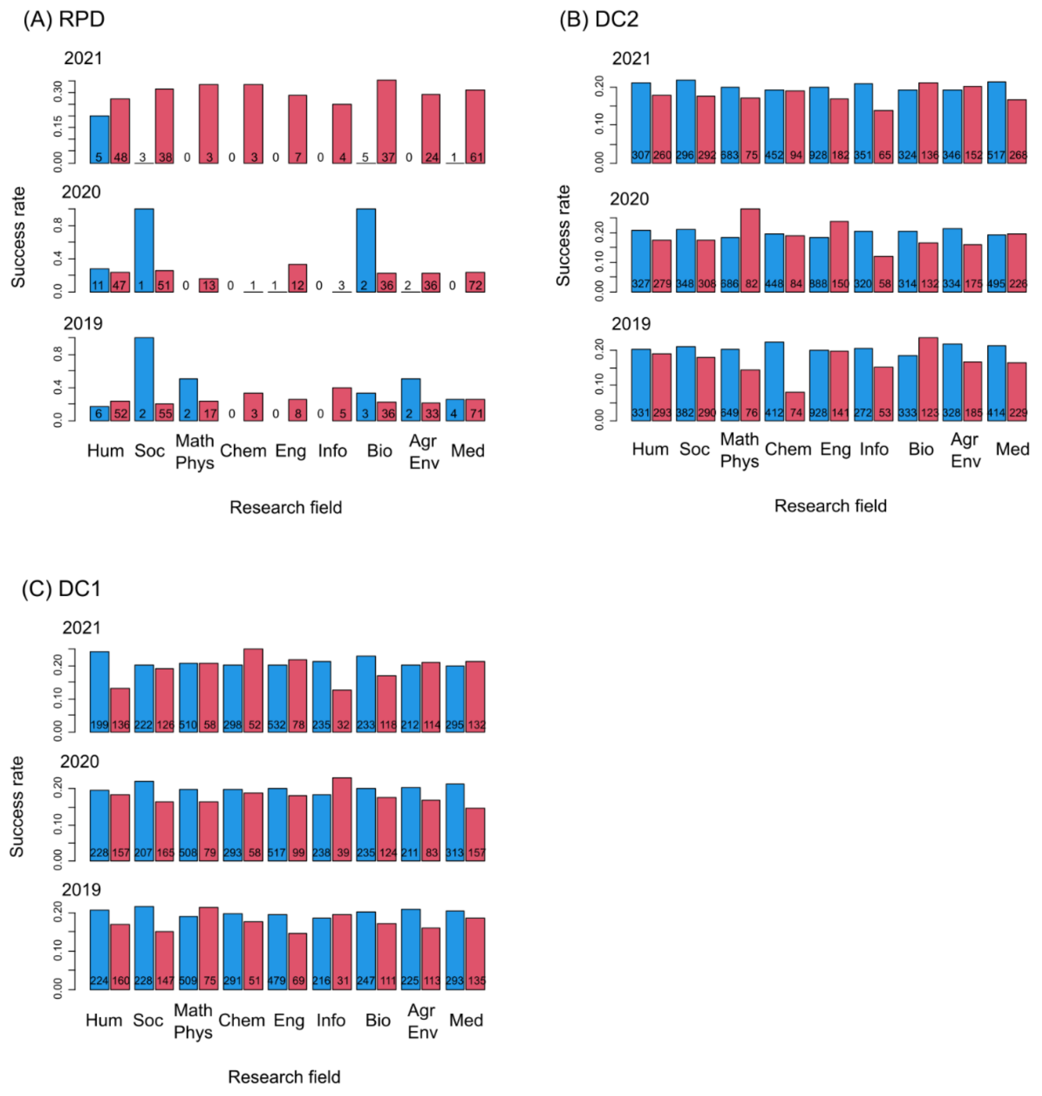

Figure S1

Gender- and research field-specific success rates of RPD (A), DC2 (B) and DC1 (C) applicants. Male and female success rates are shown in blue and red bars, respectively. Numbers on the bars represent the number of applicants. Hum: Humanity, Soc: Social Sciences, Math Phys: Mathematical and Physical Sciences, Chem: Chemistry, Eng: Engineering Sciences, Info: Infomatics, Bio: Biological Sciences, Agr Env: Agriculture and Environmental Science, Med: Medicine, Dentistry and Pharmacology.
