# Supplementary material for: Male applicants are more likely to be awarded fellowships than female applicants: A case study of a Japanese national funding agency
Source: PLoS One. 2023 Oct 25;18(10):e0291372. doi: 10.1371/journal.pone.0291372 (PMC10599527; doi:10.1371/journal.pone.0291372)
Supplement: Attachment — Submitted filename: 2023.05.02_responses.docx [file pone.0291372.s003.docx]

**Responses to the comments**

We are grateful for the constructive and helpful comments by the reviewers, and have revised the manuscript based on the comments. Below we describe our responses. The original comments are shown for reference (in blue and boxed), followed by our responses. In addition to the revisions corresponding to the reviewers’ comments, we added updated citations and those that we had not been aware of before.

**Comments by Reviewer #1**

1. under the abstract section the purpose of the research and the research methodology should be stated explicitly. Besides, research implication/recommendation should be included.

We added one sentence that explicitly explains the methodology (i.e., the use of observational data) and the purpose of the study (i.e., description of gender gaps, not of gender biases). We added another sentence that suggests future research directions.

2. there are some paragraphs with out citation. So, they should be cited

We added a citation of the JSPS URL to the fourth paragraph of the Introduction. Because the fifth paragraph provides additional details of the JSPS Fellowships, which are available on the website, we leave this paragraph without citation.

3.under methodology section the specific methods used should be justified scientifically

We elaborated on the description and rationales of data analyses. However, to be honest, we did not fully see the point of this comment. We will appreciate more specific comments, should the reviewer still find some fault in the methodology description.

4. There is a mistake also in the referencing

We have thoroughly checked the references and made corrections.

5. the title also needs to be modified

We interpreted this comment as follows: the original title could have implied the quality difference between male and female applicants. We thus modified the title so that it objectively describes the main study finding.

6. replace 'I' with 'Researcher'

As the manuscript became double-authored during the revision, we replaced “I” with “we”. To be honest, we did not see the point of this comment. We speculate that this (use of “researcher”) might be the norm in gender science literature, but we are not very sure, because we authors are both biologists by training. We are happy to replace “we” with “researchers,” if the Editor thinks we should, as the choice of subject term does not affect the reported science. But for the very same reason, we are not sure if such editing is necessary.

**Comments by Reviewer #2**

We separate the original, single-paragraph comments into chunks below.

It is important to discuss cultural aspects based on the historical evolution of the regions. Gender discrimination is observed in studies on the role of women in science in Africa, Latin America and Southeast Asia.

We elaborated on the cultural aspects in the Introduction to better contextualize our study. In particular, we now cite studies reporting gender gaps/discrimination in non-Western countries, as well as the history of relevant legislation in Japan. Yet, we note that we made this edition to the Introduction rather than to the Discussion, so that the Discussion focuses on the implications of our findings (i.e., gender and peer review).

Another important aspect to consider is the change in gender roles and the incorporation of women into the labor market. This would enrich the discussion of the article.

To better contextualize our study, we cited the statistics on the enrollment of women in the labor market in Japan (by the same token as our response to the previous comment, we made this edition to the Introduction rather than to the Discussion). We also note that in the Discussion of the revised manuscript that the reported result is a snapshot (L337-339).

Another important aspect is the consideration of the nationality of the applicants, which can also play an important role in the decision to accept or not a proposal. If these aspects are not considered, the statistical discussion could lose impact.

We appreciate this suggestion. First, some fellowship categories are basically only for Japanese. The postdoctoral fellowships (PD, RPD and Overseas) are for those with Japanese nationality or the right of permanent residence, whereas the fellowships for students (DC1 and DC2) do not have such a nationality criterion. We added this description in the Introduction (L86, L89). Unfortunately, we do not have access to the data of applicants’ nationality, and we cannot analyze the effect of nationality. We added a sentence in the Discussion pointing out that nationality may also influence the outcome of the peer review (L336).

Another element to consider is the comparative element with other offices or institutions that grant similar financing and from other regions.

We elaborated the comparison with previous findings in the fourth paragraph of the Discussion. Unfortunately, to our knowledge similar studies are not performed in non-Western countries including Japan. We thus newly pointed out the importance to perform similar studies in diverse countries at the end of this paragraph.

End of the responses.
